# Supplementary material for: Content-rich biological network constructed by mining PubMed abstracts
Source: BMC Bioinformatics. 2004 Oct 8;5:147. doi: 10.1186/1471-2105-5-147 (PMC528731; doi:10.1186/1471-2105-5-147)
Supplement: Additional File 2 — The original results of the above study (non-essential files are deleted to keep the file size under the limit set by BMC bioinformatics). [file 1471-2105-5-147-S2.bz2 › chilibotAdditionalFile2/dip10/214ID7790364E720/html/CD9_ITGB1.html]

 


 **CD9** and **ITGB1** 
  
Found 30 abstracts in PubMed, retrieved 10.  
 

 What does Google say? 
 PDF only 
| .edu only 

---

**Interactive relationship** (e.g. stimulation, inhibition, etc)

**Non-interactive relationship** (e.g. studied together, co-existance, homology, etc.)

- Expressed proteins include  **CD9** , CD10, CD13, CD29  [ **ITGB1** ] , CD34, CD44, CD 49 d, CD 49 e, CD54, CD55, CD59, CD105, CD106, CD146, and CD166.  Ref: 11573204 J Cell Physiol, 2001
- Based on their immunophenotype, the cells are relatively homogeneous, with shared expression of integrin beta 1  [ **ITGB1** ] , the hyaluronate receptor, and the tetraspan protein  **CD9** , among other markers.  Ref: 12880371 Expert Opin Biol Ther, 2003
- PMP stimulation of ECs increased CD54 and CD63 but not  **CD9** , CD29  [ **ITGB1** ] , and CD31.  Ref: 11583705 Atherosclerosis, 2001
- It also contained signaling molecules such as Xyk Xenopus egg Src, Gq alpha, Ras, integrin beta 1  [ **ITGB1** ]  and  **CD9** .  Ref: 11861472 Development, 2002
- MLSC was able to measure antigens  **CD9** , CD29  [ **ITGB1** ] , CD36, CD41, CD42a, CD42b, and CD61 on platelets and determine an average of 2.3 x 10 5 7 x 10 4 platelets per microliter.  Ref: 11487384 Platelets, 2001
- Data obtained are presented in concurrence with Clusters of Thymic Epithelial Staining CTES classification for various monoclonal antibodies recognizing CD antigens CD1, CD1a, CD6,  **CD9** , CD14, CD16, CD29  [ **ITGB1** ] , CD30, CD32, CD44, CD45RB, CD47, CD48, CD49a, CD49b, CD49c, CD49d, CD49e, CD49f, CD51, CD53, CD54, CD56, CD57, CD63, CD85, CD95, CD98, CD102, CD103, CD106, CD109, CD146, CD147, CD148, CD151, CD152, CD158a, CD158b, CD164, CD165, CD166 and for monoclonal antibodies 1B1 G7, A4, BD46, BLTZ, HP1C5, IND.64, M72, WU947 whose specifities are not yet defined.  Ref: 12725440 Ann Anat, 2003
